# Nervous System

**Published:** 1874-02

**Authors:** James Guild

**Affiliations:** Tuscaloosa, Ala.


ATLANTA

MEDICAL AND SURGICAL JOURNAL.

VOL. XI.] FEBRUARY—1874. [No. 11.

### Original Communications.

### THE NERVOUS SYSTEM AS THE PRIMARY SEAT OF MORBID IMPRESSIONS.

READ BEFORE THE TUSCALOOSA MEDICAL SOCIETY, ON MONDAY, THE 5TH OF JANUARY, 1874.

BY JAMES GUILD, M.D., TUSCALOOSA, ALA.

The present epoch in Medical Science is replete with resources for investigation into the causes and diagnosis, and consequently, treatment of diseases. The microscope is now accessible to almost every student, and the chemical analysis of the blood and secretions of the human system is now more generally resorted to in our investigations than in any preceding period in the history of medicine; based upon these advanced methods of inquiry, new theories of the causes of some of the longest known and most familiar diseases have been reared. The proximate causes of the various fevers, of Asiatic cholera, of syphilis, of gonorrhœa, of measles, variola, vaccinia, and of nearly every familiar disease have, by chemical analysis and the use of the microscope, been professedly placed beyond the eye of the investigator, and upon these recently discovered causes have been founded new theories and new methods of practice. Old familiar theories have been disproved or abandoned; old land-marks have been destroyed or ignored, and the innovations of our day have arisen in their stead and been received with wonderful avidity. Is it not to be feared that much error may creep in and produce disastrous results when practically applied; much that is false has undoubtedly been foisted upon the unthinking portion of the profession. The microscopist and analyst have presumed to speak *ex cathedra*, and in many instances none dare dispute their dicta without the risk of incurring the appellation of old fogies, who are opposed to the progress of scientific truth, and wedded to the idols of their fathers. No one can deny that recent investigations have not only directly contributed a vast amount of invaluable knowledge to the science and art of medicine, but have spoken the *open sesame* to the hitherto unopened gates of truth and pointed out its devious pathways, and at the same time exposed to view errors and false theories which for centuries had encumbered scientific progress. Admitting that they have earned a high niche in our professional temple, and yielding to them the rich reward they so justly merit, we must not too eagerly and blindly indorse their dogmas and practically accept all their teachings; for upon these beautiful plausibilities we are liable to base a false and detrimental practice. The old fathers in medicine were all *humoralists*. The peccant humors of the blood had to be expelled in disease, one way or another—no matter how, so they were gotten rid of—before health could be restored. Never since the days of Cullen has humoralism held such sway over the medical mind as now. I am glad, and thank Heaven that I have the privilege of standing here to-night, before these learned Doctors, as the advocate of great scientific truths promulgated more than half a century ago, during my pupilage at the Pennsylvania University, by such men as Physick, Chapman, Cox, Dorsey, Dewees, Hare and James, who are all dead and gone, and all renowned Humoralists.

In this practical age the pathologist and the physiologist pursue their studies, as one engaged in the exact sciences, and deal too much in the material compounds, often losing sight of the intangible, subtile agencies of life, either morbid or healthful. The chemical laboratory is appealed to and confidently relied upon, forgetting that the laboratory is the work-shop wherein they labor. In vital chemistry there is much that is so incomprehensible to our minds, that we cannot pursue its study with the satisfactory results which are attained in the study of material chemistry. Reason and philosophy have approached the precipice, looked over and retired.

In spite of all the pride of reason and philosophy the nervous force, the nervous fluid, which regulates and governs all the functions of life, still remains unexplained and clouded in vague hypothesis. It is a subject that does not admit of demonstration. We simply know that it exists and can perceive its effects and influences; but how it operates is a mere matter of speculation, and has given rise only to contradictory theories. It is a subject we cannot clearly reason upon; yet to the nervous system we must refer the controlling power of life in disease and in health. To it belongs the great mystery of medicine.

The scalpel cannot dissect and expose its wonderful operations, neither can the microscope peer into its mysteriously hidden functions. Its essence is beyond our philosophy. The physician, the minister and interpreter of nature, can act and understand in as far as he has either in fact or in thought observed the order of nature; more he can neither know nor do.

Aristotle has said that "the investigation into a final cause is a divine and not a human science, more entitled to honor, for that which is most divine is most worthy of honor." Without sacrilege, I think I might say that this branch of the science of medicine possesses some of the characteristics described by Aristotle, and though beyond our ken, should be esteemed most worthy of honor and consideration. Though it may be as hidden as the final cause, much can be learned by careful study and observation. Experience, the greatest of all teachers, can furnish us with lessons most valuable, and can store our armamentarium with weapons of defense, and with knowledge how to use them. Vitalism, in contradistinction to humoralism, is that branch of study to which we should turn our attention. Latterly the doctrine of vitalism has, I think, fallen into disrepute with some of our most laborious teachers, and the vital force has been overlooked in the therapeutical application of remedies.

The fashionable term of "*blood poison*" (an unmeaning term, in many instances) has become the stereotyped explanation of the nature and cause of almost every disease; the nervous sytem, acting in the capacity of asecondary and subordinate agent; so the cleansing of the blood has become the chief object of medication, overlooking, in their efforts, the important functions of the nervous system. What thoughtful, candid mind, that has studied the profession with care and application, can assign such a position to the nervous system. It is the key-stone of the arch; it is the head and front—the controlling principle, the animus of life. Is not, then, this blood poison the effect, not the cause of disease, in numerous instances, and should not our remedies be addressed to the vital elements of the nervous system and to the chemical constituents of the blood? Nothing can be more foreign to my purpose than indiscriminate denial and censure of the labors of those who are industriously investigating diseases according to the humoral theory, as I have stated above, they deserve and will certainly receive great praise, and their names will remain with us as household words. Prof. Salisbury has, particularly on this side of the Atlantic, given an impetus to research and investigation that will secure for him a high and honorable position in the profession. Even if he does not, in his investigations, establish true doctrines, his labors will not be in vain altogether, for they lead us to search elsewhere for the truth. The writings and lectures of Prof. Salisbury have excited considerable interest and deservedly so, but they are to be regarded valuable rather by exciting discussion than for the soundness of their philosophy. With a thorough appreciation of all earnest men, even when their faith is questionable; and thoroughly recognizing the earnestness of Prof. Salisbury, we must, however, be inclined to consider him an enthusiast, whose mind often overleaps the truth. For every disease he discovers the cause in the blood. Animalculæ and vegetable fungi of some description or other, are found, and the most minute description and classification are furnished by the learned professor. His boasted discoveries are questioned by many, some of whom can claim more skill and knowledge in microscopy, and who assert that in some instances, he has mistaken the blood corpusles and other normal constituents for his favorite infusoria and fungus parasites or cryptogamia. This theory which so entirely engrosses Prof. Salisbury is not new.

Prof. J. K. Mitchell, in 1846, advocated the cryptogamic theory of malarial fevers; in his lectures on fever, and even years before that period published a paper on the subject. In Brussels, Prof. Morseu, in 1843, adopted similar views, and on one occasion warned a pupil of his against sleeping in his room with a small quantity of mud and water he had taken from a neighboring marsh, containing cryptogamia about the period of functification, for the purpose of microscopic examination. The pupil disregarded his preceptor's injunction, and as he asserts, contracted an attack of intermittent fever. But these facts are not sufficient to establish the theory. These animalculæ and vegetable fungi—these germs of extraneous organism may be found in the human blood and secretions; but, as I have already stated, they may be the effects, not the cause of disease; or they may be simply coincident or coexistent with disease, without any causative influence whatever. The crucial test would be to inoculate a healthy individual with these germs taken from the affected person. Then if a similar disease is reproduced in the healthy individual, the theory is maintained; if not, the beautiful fabric falls to the ground. Who, for an instant, would contend that sulphate of quinine, which is universally admitted to be the most efficient remedy in intermittent and remittent fevers, and for all malarial affections, enters the blood and chemically decomposes and destroys the poison which is supposed to be lurking there as the cause of these diseases. The absurdity of such a proposition is too evident to admit of inquiry. Yet the present humoralism —the fashionable and favorite theory of blood-poisoning—spreading and ramifying, as it now does, throughout the researches of pathologists, will certainly lead to equally absurd results in its practical application. If the theory be false the practical application cannot be true; and if the practical application cannot be made useful and beneficial the theory should be abandoned as worthless. False doctrines will most assuredly lead to false practices. By the logic of induction we know that under the same circumstances, in the same diseases, and from the same causes, the same effects must result. Induction is the true logic of all physical sciences, and upon it our own science must rest to be sustained. We know that under certain circumstances, certain diseases produced by certain causes, are cured by certain remedies. This, then, is sufficient. The subtle agent that produces disease is counteracted, and the disease is cured. Why go farther? Because cryptogamic fungi are found in swamps and marshes, and also found in the human blood, does it follow that these fungi produce swamp-fevers?

Notwithstanding the long time that has been devoted to the study of fungi, there is no class of organized structure so little known. Their microscopic character, their abnormal growth, and their multifarious powers, have baffled the researches of the closest observers. Even at the present day, with all the lights of modern science, the improved means of research, and the varied observations of zealous students, the larger proportion of the microscopic forms are but imperfectly understood. These remarks apply with equal truth to animalculæ. The microscopic germs of animal and vegetable life pervade all nature. The air we breathe, the water we drink, the food we eat, swarm with myriads of cryptogamic and organic germs. Every solid and every fluid in the human system has its living parasitic tenants; yet our knowledge of them is so limited and imperfect that there can be no good reason for connecting their existence with disease at all. Closely connected with the teachings of the animal and vegetable germ theory of the causes of disease is the chemical theory. The advocates of both are searching in the blood and secretions for the causes of disease. Both, in their nosology, make blood diseases constitute the largest class. Certain maladies, commonly called blood diseases, are most promptly relieved by remedies which act specifically upon the nervous system. Hence it may be asked if there is not good reason to revise some of the favorite doctrines of the present day. When vascular engorgements and congestions are more under the control of the neurotic remedies than the lancet, it will afford Pathologists an ample apology for reconsidering certain dogmas hitherto well received; and it may be expected that better discrimination will be made in certain diseases between cause and effect. Fevers, local congestions and other forms of disease affecting the circulation of the blood, secretion, absorption and nutrition may be traced to causes acting primarily on the nervous system—the changes resulting to the fluids and solids taking their place in the category of secondary effects. Two diseases from this numerous class—gout and rheumatism—are ascribed essentially to the same causes which were assigned to them by the older writers in medicine. The unmeaning and inappropriate terms, gout and rheumatism, originated with the ancients, and are derived from their ideas of the etiology of the two diseases. The terms remain the same, and their etiology is very nearly, if not quite, the same at the present day. The old fathers believed that in gout the peccant humors of the system were distilled drop by drop into the joints. We of the present time believe that uric acid is the peccant humor of the ancients, and that it is deposited in the joints in the form of urate of soda, etc. The old authors believed that in rheumatism there was a rheum—a flow of liquid, of course peccant and offending, which permeated the system. Authors of our day believe that this rheum is lactic acid. With all of our vaunted improvements in medicine, with all of our boasted means for research and investigation, how little have we advanced in the etiology of these two diseases. Can it be that their real causes were known to the ancients, and that nothing farther remained for succeeding ages to learn; or is their etiology better understood by us of the present day? I am decidedly inclined to think that neither the ancient nor modern physicians have discovered their causes. If uric and lactic acids are the causes, why has not the alkaline treatment succeeded in curing our patients? If these causes are so palpable, why do they baffle all of our efforts to remove them? Dr. Fuller, of St. George's Hospital, London, in testing the therapeutic effects of carbolic acid recently, has discovered that it was a powerful agent in cleansing the urine of the urates, or lithates, as they were formerly called. It will even operate with certainty when moderate doses of the alkalies have failed altogether in checking the deposits. Having remarked the uniformity of the action of the carbolic acid in cleansing the urine and in checking the deposits of urates, he concluded that it might control the formation of uric acid, and administered it in full doses in cases of gout. It cleansed the urine, but did not modify the gouty action in the least; indeed, had no effect whatever upon the disease. The same result followed its use in rheumatism. And so it is with all of our remedies applied in conformity with the at present accepted theory of the humoralists—that gout and rheumatism are blood diseases, and to cure them we must cleanse the blood and secretions of peccant and offending impurities, which fail of elimination in the natural way. Gout and rheumatism are paroxysmal diseases, and nothing more can be claimed for our remedies than that they relieve the severity or shorten the duration of the paroxysms. In my long experience I have found no remedy capable of radically curing these diseases. My deceased son, Dr. LaFayette Guild, who was a great sufferer with these diseases, and fell a victim to them, diligently searched for that great boon, but failed in finding it. His views on these diseases, as well as most of the suggestions in this paper, coincided with my own. I cannot believe that the etiology of gout and rheumatism will ever be developed on purely humoral or chemical principles. The humors—to use the parlance of olden time—may contain peccant matter; the blood may contain uric and lactic acids; the secretions may be too abundant or too scanty; the liver and kidneys and all the secretory organs may act abnormally and morbidly, but it is not in these fluids, nor in the products of these secretions, that we must search for the morbific cause. They may be the effects—they may be the results of the cause of disease; but I cannot believe them to be the cause—*causa vera*—the veritable materies morbi. Cannot these extraneous morbid products in the blood and the secretions be the result of morbidly interrupted nervous force? In other words, is it not the consequence of perverted nervous action? In the electricity produced by chemical action would we seek to find the cause in the chemical products, or would we not rather direct our attention to the galvanic battery? In the decomposition of chemical compounds by electricity, and the formation of other compounds, we readily perceive that the electricity is the cause of the changes that are going on, and to correct or remove these changes we must remove the galvanic current. So in many diseases—among which we may class gout and rheumatism—the cause of the decomposition of old compounds and the formation of new substances, which are extraneous and foreign in health, is not in the chemical action which is taking place in the blood and secretions, but the cause exists in the nervous centers, and to correct or to remove that cause we must address our remedies to the nervous centers. The production of secretions is influenced by the condition of the nervous system. The quantity as well as the quality of the glandular secretions are, in a very great degree, controlled by impressions primarily made upon the nervous system. All healthful secretion follows the healthy normal condition of the nervous system. Any variation from health, any morbid or abnormal impression upon the nervous centers, deranges and diseases the secretory functions. A most distinct instance of such nervous influence is shown in cases of secretion of the earthy phosphates by the kidneys, after injury of the spinal cord. The quality and quantity of the various secretions are influenced by the mental condition of the individual; as, for instance, the abundant flow of saliva at the thought of food; the excessive secretion of urine in hysterical paroxysms; the tears excited by sorrow or excess of joy; the perspiration by terror; diarrhœa and deuresis in the young soldier under the influence of timidity on the eve of battle. The quality of a secretion being affected by mental impressions is illustrated in cases given by Carpenter, in which, through grief or passion, the secretion of milk from the mother is so changed as to produce irritation in the alimentary canal of the child, or even death.

The mode in which the nervous system influences respiration, circulation, digestion, nutrition, secretion—indeed, how it influences and controls all of the functions of life, both in health and disease, is not known. The generation of lactic acid in rheumatism, and the generation of uric acid in gout, are known only as results. How these results are brought about by the nervous system is as hidden and as mysterious to the pathologist as the decomposition of chemical compounds by electricity is hidden and mysterious to the chemist. Our errors in the etiology of gout and rheumatism have led us into erroneous practice in the treatment of these diseases. The inefficacy and futility of our remedies in establishing radical cures are proverbial, and where can we find two diseases for which such a multitudinous array of boasted remedies has been applied? Every old woman can prescribe her sovereign remedy; at every cross roads we find a *certain cure* for rheumatism and gout. It may be laid down as an axiom, that when everybody has a remedy for a disease, that disease is not understood, and therefore improperly treated by the profession. And it is in these very diseases that the quack, the charlatan or the vendor of patent nostrums finds the most fruitful field for his imposition and humbuggery. None can deny that humoralism has added much to the advancement of our profession. But what would have been our advancement if the same energy, talent, genius and patient study had been given to investigation into the hidden treasures of vitalism. Although much remains to do, much also has been accomplished, and whatever be the ultimate result, it is satisfactory to feel that we are advancing—every step bringing us nearer to positive knowledge, and that every addition to our means of research and investigation, whether in vitalism or humoralism, is leading us nearer to true and correct principles.

